# Neutrophil-to-lymphocyte ratio and CRP-to-albumin ratio in the prediction of catheter-related bloodstream infection among maintenance hemodialysis patients: a synergistical optimization algorithm

**DOI:** 10.3389/fmed.2025.1612057

**Published:** 2025-09-04

**Authors:** ZhiPeng Zhao, Shuang Yuan, XiuLi Zhang, Hang Li, XiaoYing Liu, LiHong Zhang, Tao Wang

**Affiliations:** ^1^Graduate School of Hebei Medical University, Shijiazhuang, China; ^2^Department of Nephrology, The First Hospital of Hebei Medical University, Shijiazhuang, China; ^3^Department of Nephrology, The Second People's Hospital of Shenzhen, Shenzhen, China; ^4^Department of Nephrology, Peking Union Medical College Hospital, Beijing, China; ^5^Hemodialysis Center, The First Hospital of Hebei Medical University, Shijiazhuang, China

**Keywords:** maintenance hemodialysis, neutrophil-to-lymphocyte ratio, CRP-to-albumin ratio, catheter-related bloodstream infection, central venous catheter

## Abstract

**Background:**

An improved prediction method concurrently using neutrophil-to-lymphocyte ratio (NLR) and CRP-to-albumin ratio (CAR) was herein developed for catheter-related bloodstream infection (CRBSI), which is a potentially life-threatening complication in maintenance hemodialysis (MHD) patients.

**Methods:**

In a multicenter retrospective cohort study, MHD patients using central venous catheter in the past 3 years were divided into infected and uninfected groups by the events of CRBSI. Inter-group difference was examined by either *t*-test or chi-square test. Positive findings were further stepwisely explored for independent effect, diagnostic efficacy and synergistic action on the CRBSI by binary logistic regression analysis, receiver operating characteristic (ROC) curve and multiple dimensionality reduction (MDR) method, respectively. Findings up to the ROC curve were separately validated in a second cohort, which was then incorporated for the testing of MDR method.

**Results:**

Eligible patients with and without CRBSI were 140 and 249, respectively. Hemoglobin (Hb), NLR, CAR, cholesterol, ferritin, and catheter type showed major inter-group differences and their independent effect on the CRBSI was confirmed by the regression analysis. These findings were literally validated in the second cohort. The ROC curve subsequently yielded a cutoff value of 6.115 for the NLR and 0.345 for the CAR. Accordingly, patients simultaneously manifesting higher NLR and CAR had the utmost risk for CRBSI as confirmed by the MDR method and *vice versa*.

**Conclusion:**

As such, NLR and CAR are inexpensive, replicable and easily measurable hematological indices for the prediction of future onset of CRBSI in asymptomatic patients, with better applicability and accuracy upon concurrent use.

## Introduction

Infection is a well-recognized complication and the second leading cause of hospitalization and death in maintenance hemodialysis (MHD) patients (1, 2). In this regard, vascular access-related bloodstream infection (BSI) manifested 7.7 episodes per 100 patient-years, respectively rendering hospitalizations and mortality rate of 48.0 and 1.6% (3). In fact, majority of the above said BSI occurred in patients dialyzing with central vein catheter (CVC) (3). Centers for Disease Control and Prevention (CDC) had recognized this important clinical consequence and issued report to lower the infection rates, especially the catheter-related BSI (CRBSI) (4). However, CRBSI continued to occur at unacceptable rates in the United States, possibly because of additional factors not addressed by the CDC's core interventions (5). Hence, there is a clear need for a simple and easily measurable index to predict future onset of CRBSI in asymptomatic patients as part of the prophylactic measures.

Neutrophil-to-lymphocyte (NLR) is a surrogate marker of systemic inflammation and endothelial damage, and has been associated with adverse outcomes in a range of diseases (6). Previous studies have shown that NLR may predict early occurrence of nosocomial infection and infection-related hospitalization in patients with cirrhosis (7) and end stage renal disease (8), respectively. Moreover, a recent report highlighted the use of NLR in identifying and predicting complications associated with hemodialysis and peritoneal dialysis, including cardiovascular disease and infection (9). In addition, serum C-reactive protein (CRP)-to-albumin ratio (CAR) is also a well-known indicator of infection (10).

In this study, we started with the determination of prospective association between the above said ratios (NLR/CAR) and CRBSI in a cohort of Chinese MHD patients, and proceeded to validate the findings in a second cohort. Eventually, we wrapped up with configuring an improved prediction method by concurrently using the relevant ratios, with regard to having better applicability and accuracy.

## Methods

### Study design

This work was a multicenter consecutive retrospective cohort study and the lead investigation was carried out at the Department of Nephrology in the First Hospital of Hebei Medical University, with peer collaboration from two other major domestic hospitals. Data were retrieved from the medical charts of hemodialysis patients using CVCs (the exploration cohort) between June 2021 and May 2024. Within 6 months thereafter, the findings were used for *post-hoc* test of CRBSI in similar patients from a third hospital (the validation cohort). Demographic characteristics and clinical information including comorbidity and medications were collected. Hemodialytic catheters were placed at either the femoral vein (untunneled temporary ones) or external jugular vein (tunneled long-term ones) by the Seldinger technique. Specific operation procedures of the ultrasound-guided cannulation and aseptic measures had been described in details (11). Indwelling time was recorded since the catheters were left in place and divided into two data sets by whether it exceeded 28 days (12).

The subjects included our in-center MHD patients and those referred to us seeking treatment of CRSBI, repairment of displaced catheter cuff, angioplasty of central vein stenosis or removal of catheter upon a functionally accessible fistula. Of note, the referred patients with CRBSI were usually from primary care settings or dialysis clinics (especially in the low-resource regions). They were further divided into the infected and uninfected groups based on the CRBSI development within the above-mentioned study period.

### Patient selection

Patients who met the following criteria were included: (i) aged >18 years with end stage renal diseases, (ii) regular hemodialysis for three times per week with a 4-h session each. Especially for the infection group: (iii) arrived within 24-h (in-center patients) or 48-h (referred patients) after indicatives of infection without the use of antibiotic, (iv) CRBSI diagnosed according to the relevant criteria issued by the US CDC (13): causative pathogen identified from peripheral blood and/or catheter tip, (v) absence of hypotension and complications such as serious cardiac, cerebral, or hepatic diseases. It should be specified that “indicatives of infection” were basically fever and/or chill according to the NNIS definition of CRBSI (14). Patients not compatible with these criteria were excluded.

### Hemodialysis protocols

The dialysis prescription was made according to the KDIGO guidelines, as in our previous report (15). Anemia, hypertension and hyperphosphatemia were managed by standard protocols, whereas low molecular weight heparin was used for anticoagulation.

### Laboratory tests

Venous blood including samples for culture was collected before an empirical antibiotic was given, which was usually within 1 h after hospital admission. Data of blood routine was acquired using Beckman Coulter cellular analysis system (Unicel DxH800, CA, USA). Plasma parameters were measured by using Beckman Coulter AU5800 automatic biochemical analyzer. Ferritin and intact parathyroid hormone (iPTH) were determined by Beckman Coulter automatic chemiluminescence immunoassay analyzer (UniCel DxI800). Kt/V of the hemodialysis was derived from the well-established KDOQI equation.

### Identification of causative pathogens

Blood samples were taken from the “artery” hub of the CVC and a peripheral vein simultaneously for 10 ml each and inoculated separately into individual culture bottles. The bottles were then incubated in fully automated microbial ID/AST system (DL-96A, Zhuhai DL Biotech, China) at 37 °C for 5 days. Identification of all isolates was performed using the auxiliary DL ID/AST test card. In particular, the tip of a removed catheter was also cultured and metagenomics next generation sequencing was used in case of negative blood/tip culture.

### Statistical analysis

Statistical analyses were performed using SPSS version 26.0 (SPSS, Chicago, IL, USA). All data used in the analysis were normally distributed as the significantly skewed ones were log-transformed. Student's *t-*test and the chi-square test were used for comparing continuous and categorical variables between groups, respectively. Binary logistic regression analysis was then used to examine the independent effect of NLR/CAR, if any, on the CRBSI with adjustment of potential confounding factors. The identified risk factors were further evaluated by the receiver operating characteristic (ROC) curve, which generated paired sensitivity/specificity ranking and the optimal one (cutoff value) was selected according to the Youden's index (16). Finally, interaction between risk factors that may influence the CRBSI was examined by the multifactor dimensionality reduction (MDR) method. Subjects are divided into high-and low-risk groups, using the individual cutoff value of risk factors, and the MDR method is able to detect significant inter-group difference through cross-validation and permutation testing (17). Of note, data from the exploration and validation cohorts were separately tested until the ROC curve, after which they were integrated into the MDR method. By identifying the most susceptible individuals through this method, prediction of future occurrence of infection could be accomplished, which has been effectively applied to the partitioning of our MHD patients during the COVID-19 pandemic (17). Two-sided *P* < 0.05 was considered statistically significant.

## Results

There were 140 eligible hemodialysis patients with CRBSI and 249 without it in the exploration cohort during the study period. As shown in Table 1, the infection group had more use of temporary catheter and femoral venous approach. Otherwise, there was no inter-group difference in term of gender composition, age, dialysis vintage, indwelling time, comorbidity and medications. Clinical features of the two groups were further listed in Table 2 and the significantly different ones were white blood cell, NLR, hemoglobin (Hb), procalcitonin (PCT), C-reaction protein (CRP), CAR, cholesterol, ferritin, and Kt/V. The rest of clinical parameters showed no difference between the two groups. Independent effect of the parameters with significant inter-group difference on the CRBSI (short of insertion site) was tested by the multiple logistic regression analysis and results given in Table 3. Apparently, NLR, CAR, Hb, cholesterol, ferritin, and catheter type remained as significant determinants of the CRBSI.

**Table 1:** General profiles of the exploration cohort.

	**Infection**	**Non-infection**	***P* value**
No. of patients	140	249	NA
Male (*N*, %)	77 (55.0)	149 (59.8)	0.206
Age (year)	56.8 ± 14.1	58.3 ± 15.0	0.340
**Dialysis vintage**	41.6 ± 42.1	44.6 ± 47.8	0.593
**(Month)**			
< 12 months	40	60	0.198
12 months or more	100	189	
**Catheter type (** ***N*** **)**			
Temporary catheter	83	57	**0.001**
Permanent catheter	57	192	
**Insertion site**			
Jugular vein	71	199	**0.001**
Femoral vein	69	50	
**Indwelling time**			
< 28 days	27	51	0.527
≥28 days	113	198	
**Comorbidity (%)**			
Hypertension	81.7	83.1	0.317
Diabetes mellitus	47.1	41.0	0.527
Coronary heart disease	35.8	35.0	0.213
Cerebrovascular disease	16.4	12.0	0.519
**Medication (%)**			
EPO	52.1	49.0	NA
Roxadustat	49.3	52.2	0.327
Anticoagulant	9.5	8.3	0.721

EPO, erythropoietin; NA, not applicable.

Bold values indicate statistical significance.

**Table 2:** Clinical features of the exploration cohort.

	**Infection**	**Non-infection**	***P* value**
Pre-dialysis SBP (mmHg)	148.0 ± 25.4	144.3 ± 24.0	0.152
Pre-dialysis DBP (mmHg)	83.5 ± 14.6	80.8 ± 17.2	0.133
White blood cell (× 10^9^/L)	9.3 ± 3.8	5.4 ± 2.0	**0.001**
Neutrophil (× 10^9^/L)	7.1 ± 3.5	3.8 ± 1.6	**0.001**
Lymphocyte (× 10^9^/L)	0.8 ± 0.5	1.2 ± 1.4	**0.001**
Neutrophil-lymphocyte ratio^†^	12.0 ± 8.2	4.9 ± 2.9	**0.001**
Hemoglobin (g/L)	83.1 ± 8.2	107.1 ± 8.3	**0.029**
Platelet (× 10^9^/L)	196.3 ± 78.6	198.1 ± 89.0	0.387
Procalcitonin (ng/ml)^†^	1.8 ± 2.0	0.8 ± 0.4	**0.047**
C-reaction protein (mg/L)^†^	33.0 ± 42.6	6.5 ± 10.6	**0.001**
CRP-albumin ratio^†^	0.97 ± 1.33	0.18 ± 0.30	**0.001**
Albumin (g/L)	36.4 ± 4.3	37.0 ± 4.5	0.228
Pre-dialysis BUN (mmol/L)	27.2 ± 9.0	28.3 ± 8.5	0.632
Pre-dialysis Scr (μmol/L)^†^	997.2 ± 229.2	935.5 ± 345.6	0.386
Potassium (mmol/L)	5.1 ± 0.7	4.9 ± 0.8	0.155
Calcium (mmol/L)	2.1 ± 0.3	2.1 ± 0.2	0.661
Phosphate (mmol/L)	2.0 ± 0.3	1.8 ± 0.6	0.387
Cholesterol (mmol/L)	3.1 ± 0.9	4.5 ± 1.3	**0.021**
Triglyceride (mmol/L)^†^	1.75 ± 0.92	1.75 ± 1.38	0.386
Ferritin (ng/ml)^†^	698.0 ± 92.2	397.6 ± 85.7	**0.001**
iPTH (pg/ml)^†^	280.7 ± 134.3	259.8 ± 121.3	0.991
Kt/V^†^	1.1 ± 0.3	1.3 ± 0.3	**0.001**

Results are given as mean ± SD. Differences between the groups were examined by χ^2^ test or unpaired t-test when deemed appropriate. P < 0.05: significant inter-group difference.

^†^Log-transformed values used in the SPSS analysis. SBP and DBP: systolic and diastolic blood pressure, respectively.

BUN, blood urea nitrogen; Scr, serum creatinine; iPTH, intact parathyroid hormone.

Bold values indicate statistical significance.

**Table 3:** Risk factors for hemodialytic catheter-associated BSI in the exploration cohort.

**Effect**	**Model fitting criteria**	**Likelihood ratio tests**		
	−**2 log likelihood of reduced model**	**Chi-square**	**df**	**Sig**.
Intercept	283.997^a^	0	0	–
Neutrophil-lymphocyte ratio^†^	333.20	49.20	1	0.000
CRP-Albumin ratio^†^	322.30	38.30	1	0.000
Hemoglobin	295.51	11.51	1	0.001
Cholesterol	287.14	7.21	1	0.046
Ferritin^†^	301.33	17.33	1	0.000
Catheter type	314.11	30.11	2	0.000

^a^This reduced model is equivalent to the final model because omitting the effect does not increase the degrees of freedom. The intercept in this model represents the value of the dependent variable when all independent variables are zero.

^†^Log-transformed value used in the SPSS test.

Possible diagnostic efficacy of the NLR and CAR on CRBSI was evaluated by the ROC curve, using data of the exploration cohort. It subsequently yielded, in the order of sensitivity, specificity and area under curve, 0.707, 0.775, and 0.756 for the NLR, 0.750, 0.723, and 0.772 for the CAR. Accordingly, the cut-off value for NLR and CAR was 6.615 and 0.345, respectively.

In the context of causative pathogen, positive rates of blood culture for the peripheral vein and “artery” hub were 36.4 and 46.4%, in comparison of a 59.0% positive rate for the 122 removed catheter tips. Causative pathogens in “all negative” cases were nailed down by the mNGS technique. As such, the percentage of Gram positive and negative bacteria was 60.0 and 37.1%, respectively, in addition to 2.9% of fungus.

There were 46 hemodialysis patients with CRBSI and 68 without it in the validation cohort, with same inter-group difference in the general profiles (Table 4). Significantly different clinical features were similar to those in the exploration cohort, but with comparable lymphocyte count and PCT irrespective of the CRBSI (Table 5). Likewise, independent effect of the NLR, CAR, Hb, cholesterol, ferritin, and catheter type on the CRBSI was confirmed by the multiple logistic regression analysis (data not shown). Of note, 23 patients had a NLR ratio higher than 6.615 and 14 of them were in the infection group. This composition produced a sensitivity and specificity of 71.8 and 72.1%, respectively. As for the characteristics of CRBSI pathogens, positive rates of culture were 37.0%, 41.3% and 53.8% for samples taken from the peripheral vein, “artery” hub and 39 removed catheter tips, respectively. Percentage of Gram positive bacteria was 65.2% and the rest was Gram negative ones, with absence of fungus.

**Table 4:** General profiles of the validation cohort.

	**Infection**	**Non-infection**	***P* value**
No. of patients	46	68	NA
Male (*n*, %)	25 (54.3)	36 (52.9)	0.316
Age (year)	61.6 ± 12.1	60.5 ± 13.2	0.639
**Dialysis vintage**	38.0 ± 35.4	39.4 ± 35.2	0.615
**(Month)**			
< 12 months	14	21	0.108
≥12 months or more	32	47	
**Catheter type (** ***N*** **)**			
Temporary catheter	26	20	**0.001**
Permanent catheter	20	48	
**Insertion site**			
Jugular vein	25	50	**0.045**
Femoral vein	21	18	
**Indwelling time**			
< 28 days	10	16	0.505
≥28 days	36	52	
**Comorbidity (%)**			
Hypertension	89.1	86.8	0.513
Diabetes mellitus	47.8	42.6	0.728
Coronary heart disease	39.1	35.3	0.622
Cerebrovascular disease	15.2	16.2	0.823
**Medication (%)**			
EPO	67.4	67.6	0.811
Roxadustat	54.3	57.3	0.534
Anticoagulant	15.2	17.6	0.326

EPO, erythropoietin; NA, not applicable.

Bold values indicate statistical significance.

**Table 5:** Clinical features of the validation cohort.

	**Infection**	**Non-infection**	***P* value**
Pre-dialysis SBP (mmHg)	139.5 ± 13.8	140.5 ± 24.0	0.471
Pre-dialysis DBP (mmHg)	80.1 ± 12.3	82.6 ± 11.9	0.433
White blood cell (× 10^9^/L)	8.9 ± 3.1	5.2 ± 2.0	**0.001**
Neutrophil (× 10^9^/L)	7.0 ± 4.3	4.0 ± 1.9	**0.001**
Lymphocyte (× 10^9^/L)	0.8 ± 0.6	0.9 ± 1.1	0.131
Neutrophil-lymphocyte ratio^†^	11.5 ± 6.5	5.8 ± 4.6	**0.016**
Hemoglobin (g/L)	92.7 ± 8.7	111.2 ± 11.6	**0.001**
Platelet (× 10^9^/L)	183.4 ± 85.6	297.2 ± 76.9	0.239
Procalcitonin (ng/ml)^†^	1.9 ± 1.3	1.2 ± 1.6	0.106
C-reaction protein (mg/L)^†^	49.5 ± 35.8	7.6 ± 10.2	**0.027**
CRP-albumin ratio^†^	1.52 ± 0.27	0.25 ± 0.30	**0.001**
Albumin (g/L)	35.1 ± 6.0	39.5 ± 6.7	0.068
Pre-dialysis BUN (mmol/L)	23.6 ± 8.0	21.1 ± 7.5	0.406
Pre-dialysis Scr (μmol/L)^†^	777.0 ± 385.9	823.2 ± 273.7	0.461
Potassium (mmol/L)	4.6 ± 0.8	5.0 ± 0.9	0.115
Calcium (mmol/L)	2.2 ± 0.2	2.1 ± 0.4	0.551
Phosphate (mmol/L)	1.9 ± 0.8	2.0 ± 0.7	0.238
Cholesterol (mmol/L)	3.8 ± 1.2	4.3 ± 1.0	**0.031**
Triglyceride (mmol/L)^†^	1.3 ± 0.8	1.5 ± 0.8	0.137
Ferritin (ng/ml)^†^	498.6 ± 123.8	232.3 ± 115.2	**0.019**
iPTH (pg/ml)^†^	303.2 ± 150.6	275.1 ± 236.8	0.403
Kt/V^†^	NA	NA	NA

Results are given as mean ± SD. Differences between the groups were examined by χ^2^ test or unpaired t test when deemed appropriate. P < 0.05: significant inter-group difference.

^†^Log-transformed values used in the analysis.

SBP and DBP, systolic and diastolic blood pressure, respectively; BUN, blood urea nitrogen; Scr, serum creatinine; iPTH, intact parathyroid hormone.

Bold values indicate statistical significance.

**Figure 1:**
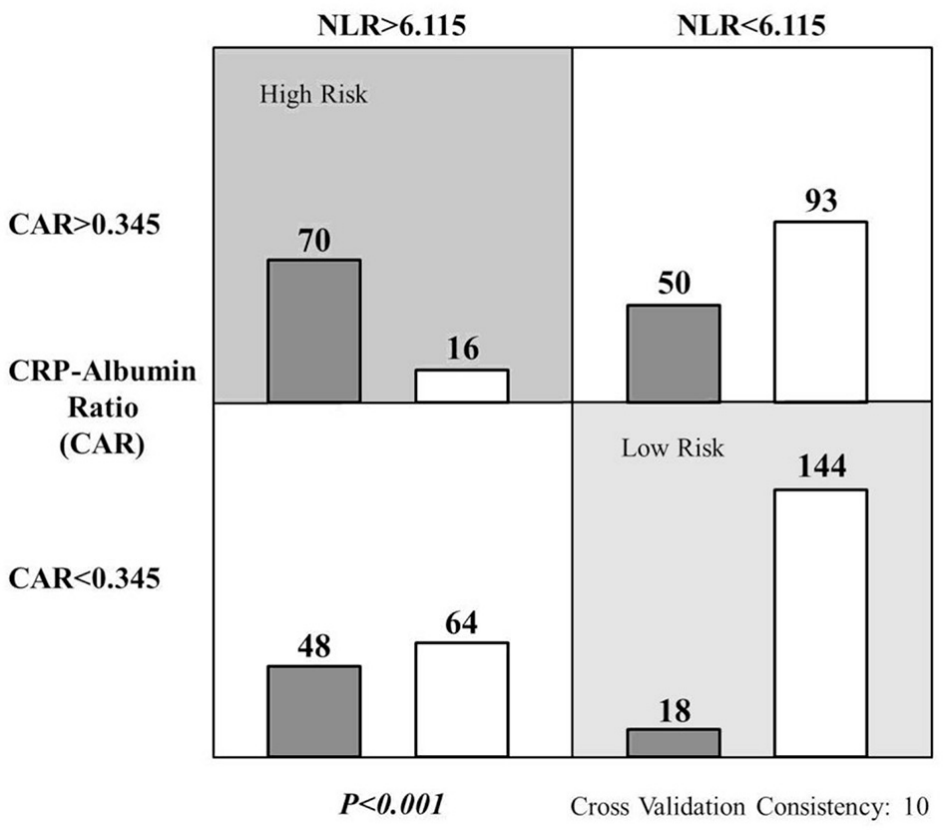
Significant interactive actions of the NLR and CAR on the risk of CRBSI in the studied MHD patients. Left and right bars in each cell represent patients with and without the CRBSI, respectively. Dark gray cell indicates higher risk of infection and the light gray one for lower risk.

After data of the two cohorts were pooled together, the patients were ulteriorly divided into four subgroups using the cutoff values of NLR and CAR and subjected to the testing of MDR method. It was clear that patients simultaneously manifesting higher NLR and CAR had the utmost risk for CRBSI and *vice versa* (Figure 1).

## Discussion

Today NLR is widely used across almost all medical disciplines as a reliable and easily applicable marker of immune response to various infectious and non-infectious stimuli. However, its application in the CRBSI among MHD patients has been sparsely explored. Likewise, this situation is also true for the CAR, with paucity of related reports. We in this work found a predictive role of NLR or CAR in the CRBSI, whereas concurrent use of these ratios was more accurate compared to a separate evaluation.

Conserved by evolution, neutrophilia and lymphocytopenia are acute dynamic changes that reflect the natural physiological response of circulating leukocytes to stress, injury, trauma, major surgery and infection (18). Neutrophils, although more numerous, are relatively hypofunctional and absolute lymphopenia is common during sepsis as highlighted in a latest work of the NEMJ (19). The NLR is hence the optimal expression of the balance between innate (neutrophil) and adaptive (lymphocyte) immune response, as it integrates information regarding these two distinct leukocyte subtypes. Indeed, the NLR has been shown to be significantly associated with inflammation and death (20), and can predict mortality in patients undergoing dialysis (21). A normal range of NLR is reportedly between 1.0 and 2.0 and the values higher than 3.53 and below 0.78 in adults are pathological (22), with a value above 11.7 significantly associated with mortality in COVID-19 infection (23). Determination of the NLR cut-off value was explicitly proposed in a study of infection and hemodialysis catheter survival (24), as such a value was essential for constructing management algorithms. Upon and beyond these understandings, we came up with not only a specific value, but also an algorithm, namely, a pragmatic method for predicting future onset of CRBSI prior to the clinical diagnosis.

We detected a positive correlation between NLR and CRBSI in MHD patients using multivariable regression. This correlation likely stemmed from the role of neutrophils and lymphocytes in the immune response: neutrophils are crucial in the initial defense against infections, while lymphocytes are involved in the adaptive immune response. An elevated NLR indicates a heightened inflammatory state, which is known to predispose patients to infection-related catheter exchange (24) or pneumonia in the MHD population (25). Reportedly, there is also a correlation between serum PCT concentration and NLR values in patients with infectious diseases (26), with NLR presumably having higher predictive efficiency than PCT in early sepsis (27). However, PCT is the predictor in a full-swing infection but not in an early one (28) and this may explain its absence of difference in the validation cohort. More realistically, whether protocol using PCT was generalizable to low-resource settings was recently questioned (29). On a practical level, a large portion of the patient population received long-term dialysis in local hospitals or clinics and it was in these settings that complications including the CRBSI occurred (30). Therefore, a straightforward and cost-effective biomarker with predictive attributes is needed in these primary care settings, especially the low-resource ones. *A priori*, these considerations qualified the NLR as an excellent one.

Yang et al. (31) has previously reported elevated NLR for the early diagnosis of CRBSI in MHD patients. Their work also yielded a cut-off value of 4.485 for NLR and 100% positive rate of peripheral blood culture. It was unusual to find that their conclusion was based on mere *t*-test between the infection and no-infection groups, without taking into account cofounding factors. In this regard, we are more inclined to trust the multivariate analysis as in our recent work establishing susceptibility model for COVID-19 infection in the MHD population (17). Secondly, a 100% positive rate of blood culture seemed far beyond our reach (32). Lee et al. (33) reported a positivity of 73.1 and 89.7% with the first blood culture and first two blood cultures, respectively, obtained during the 24-h period of bacteremia. As the last major divergence, predominant pathogen of CRBSI was Gram positive bacteria in our work, which was in agreement with currently known data (34).

While interpreting the relevance of NLR and CAR on CRBSI, it is important to consider the potential association between malnutrition and inflammation. Actually, malnutrition-inflammation-atherosclerosis (MIA) syndrome is an important issue in MHD patients as we have highlighted previously (15). Therefore, CAR is also a reliable risk indicator for inflammatory conditions as CRP and albumin are two major participants in the MIA syndrome. Consistently, our findings indicated that better management of Hb and cholesterol may predispose the MHD patients to lower risk of CRBSI, whereas higher ferritin levels act conversely. Obviously, anemia was associated with increased risk for CRBSI (35) and we have specified in MHD patients the role of cholesterol as a surrogate marker for nutrition (15) and participation of iron load in infection elsewhere (17, 32). The fact that long-term catheter is more resistance to CRSBI than the temporary ones is beyond all doubt (36). Finally, these major findings were confirmed in the validation cohort.

Admittedly, a single clinical variable may not be sufficient to accurately predict CRBSI. This is why we tried to configure the NLR and CAR in a role that maximum their efficacy, a task relied heavily on the MDR method. By nature, this method is a multivariate non-parametric approach that helps to simultaneously detect and characterize multiple genetic loci (designated as factor in the MDR) associated with a discrete clinical end-point. By design, effect of the factors was simplified into a “binary” way, which in turn abrogates possible interference among them. By function, this method made it possible for the analysis of small number of samples when traditional methods cannot be applied (37). By practice, performance of the MDR method in skewed distributions was superior to several current methodologies (38), including the principle component analysis which is the backbone of dimensionality reduction in SPSS (39). Taken together, the MDR method is a trustworthy tool regardless of the correlations between factors, the sample size and data distribution. In our current work, subjects were partitioned by the cutoff values of NLR and CAR (assigned as presumed “factors”), their CRBSI status presented in binary format and possible high-order interaction between these two variables successfully tested (Figure 1). Evidently, the MDR-based stratification may contribute to the clinical decision-making as a triage tool (17). As such, interventions of the CDC's collaborative prevention program (5) could be implemented more pointedly and efficaciously.

There are still some limitations in our work concerning its retrospective design, dynamic changes in NLR/CAR and our single calculation of the optimal cut-off value of NLR in a cohort with limited patient number. Otherwise, a subgroup analysis by the type of causative pathogens would have been performed for a better understanding of the predictive value of NLR and CAR in different types of CRBSI. Indeed, NLR is useful to discriminate infections caused by fungus from Gram positive bacteria in febrile patients with BSI, whereas IL-6 could break the G^+^ group from G^−^ one in these patients (40). Further work focusing on NLR, CAR, and cytokines in a prospective way and with multicenter collaboration has been undertaken accordingly.

## Conclusions

NLR is a dynamic parameter with a quick response to infections and it may reflect development of the CRBSI in the MHD patients. Combination of the NLR with CAR may further mitigate its diagnostic limitations and provide more precise information on CRBSI. Arguably, these findings emphasized the importance of identifying high-risk patients who may benefit from early therapeutic interventions.

## Data Availability

The raw data supporting the conclusions of this article will be made available by the authors, without undue reservation.
